# Dexmedetomidine in prevention and treatment of postoperative and intensive care unit delirium: a systematic review and meta-analysis

**DOI:** 10.1186/s13613-018-0437-z

**Published:** 2018-09-20

**Authors:** Julian Flükiger, Alexa Hollinger, Benjamin Speich, Vera Meier, Janna Tontsch, Tatjana Zehnder, Martin Siegemund

**Affiliations:** 1 Department for Anesthesia, Surgical Intensive Care, Prehospital Emergency Medicine and Pain Therapy, University Hospital Basel, Spitalstrasse 21, 4031 Basel, Switzerland; 2 Department of Anaesthesiology, Burn and Critical Care Medicine, AP-HP, Saint Louis and Lariboisière University Hospitals, 2 rue Ambroise Paré, 75010 Paris, France; 3 Inserm 942 Paris, Biomarqueurs et maladies cardiaques, Hôpital Lariboisière - Bâtiment Viggo Petersen, 41, boulevard de la Chapelle, 75475 Paris Cedex 10, France; 4 Department of Clinical Research, Basel Institute for Clinical Epidemiology and Biostatistics, University of Basel and University Hospital Basel, Basel, Switzerland; 5 Nuffield Department of Orthopaedics, Rheumatology and Musculoskeletal Sciences, Centre for Statistics in Medicine, University of Oxford, Oxford, UK

**Keywords:** Delirium, Dexmedetomidine, Meta-analysis

## Abstract

**Background:**

To determine the preventive and therapeutic effect of dexmedetomidine on intensive care unit (ICU) delirium.

**Methods:**

The literature search using PubMed and the Cochrane Central Register of Controlled Trials was performed (August 1, 2018) to detect all randomized controlled trials (RCTs) of adult ICU patients receiving dexmedetomidine. Articles were included if they assessed the influence of dexmedetomidine compared to a sedative agent on incidence of ICU delirium or treatment of this syndrome. Accordingly, relevant articles were allocated to the following two groups: (1) articles that assessed the delirium incidence (incidence comparison) or articles that assessed the treatment of delirium (treatment comparison). Incidence of delirium and delirium resolution were the primary outcomes. We combined treatment effects comparing dexmedetomidine versus (1) placebo, (2) standard sedatives, and (3) opioids in random-effects meta-analyses. Risk of bias for each included RCT was assessed following Cochrane standards.

**Results:**

The literature search resulted in 28 articles (25 articles/4975 patients for the incidence comparison and three articles/166 patients for the treatment comparison). In the incidence comparison, heterogeneity was present in different subgroups. Administration of dexmedetomidine was associated with significantly lower overall incidence of delirium when compared to placebo (RR 0.52; 95% CI 0.39–0.70; *I*^2^ = 37%), standard sedatives (RR 0.63; 95% CI 0.46–0.86; *I*^2^ = 69%), as well as to opioids (RR 0.61; 95% CI 0.44–0.83; *I*^2^ = 0%). Use of dexmedetomidine significantly increased the risks of bradycardia and hypotension. Limited data were available on circulatory insufficiency and mortality. In the treatment comparison, the comparison drugs in the three RCTs were placebo, midazolam, and haloperidol. The resolution of delirium was measured differently in each study. Two out of the three studies indicated clear favorable effects for dexmedetomidine (i.e., compared to placebo and midazolam). The study comparing dexmedetomidine with haloperidol was a pilot study (*n* = 20) with high variability in the results.

**Conclusion:**

Findings suggest that dexmedetomidine reduces incidence and duration of ICU delirium. Furthermore, our systematic searches show that there is limited evidence if a delirium shall be treated with dexmedetomidine.

**Electronic supplementary material:**

The online version of this article (10.1186/s13613-018-0437-z) contains supplementary material, which is available to authorized users.

## Background

Delirium is experienced in 20% to 40% of the critically ill and up to 80% of mechanically ventilated (MV) medical or surgical patients [1–4]. It is a confusional state that has been described as a transient global disorder of cognition, awareness, and attention and as such is not only challenging for the treating medical team, but also has a considerable impact on affected patients. It is associated with prolonged hospital length of stay and time on MV, deterioration in cognition, and increased morbidity and mortality causing additional health-care expenses [4–6]. Its pathophysiological mechanisms are highly heterogeneous—as perceived by the high number of risk factors [7]—and have yet to be fully understood. Imbalanced neurotransmitter systems such as the reduction of acetylcholine activity, excess of serotonin and dopamine, or the release of gamma-amino-butyric acid (GABA) seem essential. Certain hospital care characteristics (patient immobilization, bladder catheter, or sleep deprivation) further contribute to the development of delirium [8, 9].

Treatment approach with sedation offers several benefits: First, it reduces discomfort, anxiety, and stress. Second, it facilitates the daily intensive care unit (ICU) procedures for the treating nurses and physicians, providing a calm, cooperative patient who is easy to rouse and capable of communicating pain and other needs [10–12]. So far, there is no ideal sedative agent that fulfills the criteria of being cheap, rapid in onset and offset, and without local or systemic adverse effects [10].

Although the 2013 PAD guidelines suggested against the use of antipsychotics such as haloperidol for the treatment of delirium due to their side effects (e.g., extrapyramidal symptoms, neuroleptic malignant syndrome, and QTc interval prolongation), they are still commonly applied today [1, 13, 14]. Moreover, the most recent 2013 American College of Critical Care Medicine Guidelines for the management of pain, agitation, and delirium (PAD) in adult ICU patients recommend the use of a non-benzodiazepine-based sedation approach [15].

Dexmedetomidine with its broad range of effects including easily controllable sedation, analgesia, and anxiolysis still enables the caring medical team to interact with the patient. It reduces the activity while still maintaining the reactivity of neurons in the locus coeruleus. Therefore, it is an appealing alternative to traditional sedatives such as propofol and benzodiazepines [16]. As a highly selective $\propto$_2_-receptor agonist with no effect on the GABA receptor, it interacts with transmembrane G-protein-binding adrenoreceptors in the periphery ($\propto$_2A_), as well as in the brain ($\propto$_2B_) and the spinal cord ($\propto$_2C_). Inducing a sleep-like state without respiratory depression may explain the beneficial effects of dexmedetomidine [17, 18], since disturbed circadian rhythm is a known contributing factor of delirium [19]. These characteristics lead to an easily arousable, communicative, and cooperative patient and render dexmedetomidine a potential therapeutic option for the ICU delirium, in addition to its suggested use for delirium prevention [16, 20]. However, inhibition of sympathetic activity in the periphery leads to sequential decreases in blood pressure and heart rate [19], the most commonly reported adverse events associated with dexmedetomidine [21].

A review from 2013 suggested dexmedetomidine could be suitable for both prevention and treatment of ICU-associated delirium [14]. In 2014, a systematic review from Pasin et al. [20] including 14 RCTs with a total of 3029 patients demonstrated that use of dexmedetomidine for anesthesia and sedation was associated with a significant reduction of the incidence of delirium. Similar results were demonstrated in a previous review from Xia et al. where only propofol was used as a comparator [22]. Very recently, Duan et al. [23] performed a meta-analysis, showing that dexmedetomidine can reduce postoperative delirium incidence in adult cardiac and non-cardiac surgery patient. Based on fact that in the meantime a number of additional randomized controlled trials (RCTs) were published, we wanted to provide an up-to-date meta-analysis on dexmedetomidine for both treatment and prevention of ICU delirium to define areas of future research concerning ICU delirium and dexmedetomidine treatment. Randomized controlled trials (RCTs) were included, assessing the effect of dexmedetomidine on delirium in adult ICU patients. The goal was to summarize current evidence on the potential of dexmedetomidine to lower the incidence and duration of ICU delirium.

## Methods

For this systematic review and meta-analysis, we adhered to the PRISMA statement for reporting systematic reviews and meta-analyses [24].

### The literature search

Authors performed an electronic database search of PubMed and the Cochrane Central Register of Controlled Trials (CENTRAL). The detailed search strategy is available in appendix (Additional file 1: Appendix). We used standard filters to search for RCTs. The last date of search was August 1, 2018.

### Inclusion criteria

Two independent investigators (JF and AH) identified all potentially relevant studies in PubMed and CENTRAL based on a screening of titles and abstracts. Of these, full texts were obtained and reviewed for eligibility by the same two investigators. Any conflict of opinion was resolved by consensus with a third reviewer (MS).

All studies that met the following inclusion criteria were selected: (1) RCT; (2) adult (≥ 18 years) medical or surgical ICU patients; (3) sedation with dexmedetomidine versus any comparator either for prevention or treatment of ICU delirium, regardless of dose, duration, or time of administration; (4) incidence of delirium as a mandatory outcome measurement regarding incidence comparison; and (5) full text available in English. The exclusion criteria were duplicate publications, missing indication of delirium incidence, study design other than RCT, focus on withdrawal delirium, oral administration of dexmedetomidine, or dexmedetomidine administered in both intervention and control groups.

### Data extraction

Study-relevant information was extracted by two independent investigators (JF and AH) for each included RCT. Any conflict of opinion was resolved by consensus with a third reviewer (MS).

Interventional (dexmedetomidine) and control drugs, publication date, study location and date of study conduct, as well as patient characteristics, total number of patients, and conducted procedure were considered relevant for data extraction. Control drugs were divided into three groups: placebo, standard sedatives (including propofol, midazolam, and lorazepam), and opioids (including morphine and remifentanil). Delirium assessment tools (e.g., confusion assessment method for the ICU = CAM–ICU or the intensive care delirium screening checklist = ICDSC) were assessed. Furthermore, we recorded if delirium was assessed as the primary, a secondary, or no specific endpoint.

Although our inclusion criteria focused only on the incidence of delirium, we also conducted further analyses of other patient-relevant factors, such as adverse events (i.e., mortality, bradycardia, tachycardia, hypotension, hypertension, and circulatory insufficiency) and clinical outcome data (i.e., ICU length of stay, and time to extubation or duration of MV). These analyses have only an explorative character and raise no claim to completeness.

According to whether dexmedetomidine and control drugs were applied for treatment or prevention of ICU delirium, included trials were assigned to incidence comparison or delirium treatment (treatment comparison) and analyzed according to their affiliation.

For assessment of adverse events, we also considered studies reporting adverse events during anesthesia and subsequent intensive care sedation to increase the reliability of the study since only a few studies reported adverse events during intensive care sedation with dexmedetomidine.

The primary outcome measure in incidence comparison was incidence of delirium. The primary outcome measure in treatment comparison was delirium resolution.

### Risk of bias assessment

Risk of bias for each included RCT was assessed using the Cochrane Collaboration’s tool [25]. Two investigators (JF and BS) reviewed each RCT for risk of bias considering the following five key domains: (1) random sequence generation (selection bias); (2) allocation concealment (selection bias); (3) blinding of participants and personnel (performance bias); (4) blinding of outcome assessment (detection bias); and (5) incomplete outcome data (attrition bias). Each domain was judged as “low risk,” “high risk,” or “unclear risk” of bias. Any disagreement in opinion was resolved by consensus. We estimated the overall risk of bias for a RCT as low if the risk of bias was low in all key domains, as unclear if the risk of bias was unclear in at least one key domain, and as high if the risk of bias was high in at least one key domain.

### Statistical analysis

Meta-analyses were conducted using the Cochrane Review Manager 5 (RevMan5). Differences in binary outcomes were presented as risk ratios (RR) including 95% confidence intervals (CI) based on random and fixed effect models. RRs below 1 indicate that the event occurred less often in patients who received dexmedetomidine compared to patients who received a control drug. In incidence comparison, meta-analyses were performed to compare the incidence of delirium, adverse events, and clinical outcomes for each control drug separately. For the incidence of delirium, the following subgroup and sensitivity analyses were conducted: analyzing only studies with low risk of bias according to risk of bias assessment, population (i.e., cardiac surgery, MV, and non-cardiac surgery), and using a trim-and-fill method [26, 27] if there was an indication of publication bias.

Higgin’s *I*^2^ was conducted to assess the heterogeneity of the studies [28]. If a subgroup consisted of sufficient RCTs, Egger’s test was performed and funnel plot was visually assessed to determine the risk of publication bias. Symmetric statistics and trim-and-fill methods were conducted using R version 3.4.1 (meta package) following the Cochrane recommendations [29]. Furthermore, meta-analyses were conducted for the following adverse events and clinical outcomes: mortality, bradycardia, tachycardia, hypotension, hypertension, circulatory insufficiency, ICU length of stay, and time to extubation or duration of MV. If both time to extubation and duration of MV were presented, we used time to extubation data for the meta-analyses. In case no means and standard deviations (SDs) were reported (for ICU length of stay, time to extubation or duration of MV), we used medians, interquartile ranges (IQRs), or ranges to estimate these attributes, forcing us to make several assumptions (Additional file 1: Tables S1–S3) [30, 31].

Mortality rates assessed up to 45 days and the remaining adverse events were pooled regardless of the time they occurred (intraoperatively, postoperatively, during sedation or MV).

For treatment comparison, all articles reported different outcomes. Therefore, results are summarized descriptively and no meta-analysis was performed.

## Results

### Trial identification

**Fig. 1:**
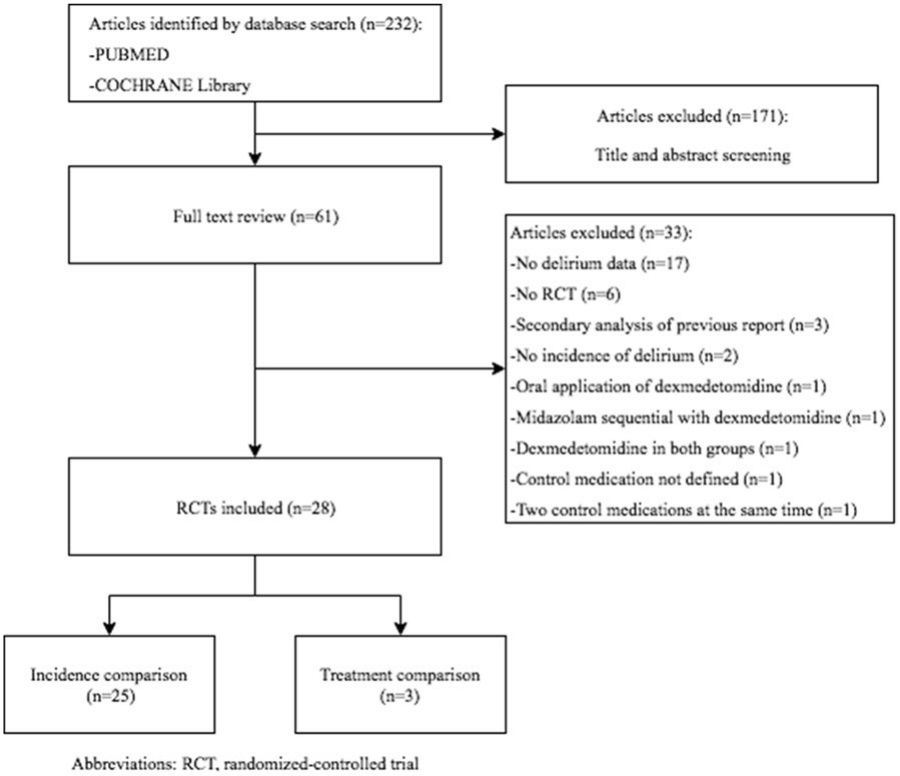
Flow diagram of the systematic search for RCTs

After excluding duplicate studies, our literature search on PubMed and CENTRAL retrieved 232 articles. By screening titles and abstracts, 171 publications were excluded at the title abstract level and an additional 33 were excluded after consulting the full text (Fig. 1). The remaining 28 articles were included and divided into incidence comparison consisting of 25 RCTs [32–56] and treatment comparison consisting of three articles [57–59] (Fig. 1).

### Study characteristics

In incidence comparison, all RCTs were published between 2005 and 2018 (being conducted between 2002 and 2017; Table 1). Two RCTs did not state study dates [39, 40]. Sixteen RCTs dealt with surgical patients, nine of which were cardiac surgery RCTs [36, 39, 41, 42, 45–47, 51, 53] and seven non-cardiac surgery RCTs, meaning microvascular free flap surgery [34], joint replacement [49] or total hip arthroplasty [54], major abdominal surgery [52], major laparoscopic surgery [55], and elective non-cardiac surgery under general anesthesia [33, 48]. The remaining nine RCTs consisted of six studies examining MV patients [32, 38, 40, 43, 44, 50] two examining noninvasively ventilated patients [35, 37] and one where dexmedetomidine was used for nightly sedation [56]. Applied procedures for administering intervention and control drugs were highly heterogeneous (Table 1). Comparators were placebo in eight RCTs [33–35, 45, 48, 51, 55, 56], propofol in six RCTs [42, 46, 47, 52–54], midazolam in two RCTs [37, 50], and lorazepam [32], morphine [41], and remifentanil [36] in one RCT each. All of these RCTs used a two-arm design. Two RCTs compared dexmedetomidine sedation to a sedation regimen of either propofol or midazolam [43, 44]. Two RCTs used a three-arm design comparing dexmedetomidine to midazolam and propofol [39, 40]. One RCT used a two-arm design comparing dexmedetomidine to placebo, but did not report overall results. Instead, only results for the two subgroups “normal-aged” patients and patients with amnestic mild cognitive impairment (aMCI) after joint replacement were reported [49]. One article consisted of two separate RCTs comparing dexmedetomidine to midazolam and propofol, respectively [38]. The total number of patients in incidence comparison was 4975. The used tools for delirium assessment are listed in Table 1. In treatment comparison, all RCTs were published between 2009 and 2016 (study dates ranged from 2005 to 2013; Table 1). Study populations of all RCTs consisted of patients with agitated delirium. Two RCTs dealt with MV patients in whom extubation was considered unsafe because of agitated delirium [57, 59]. One RCT examined patients presenting with delirium following extubation failure after cardiac surgery [58]. Interventional and control drugs were administered according to the desired RASS score [57, 59], except for one RCT, where they were administered according to blood pressure and heart rate [58]. Comparators were placebo [57], midazolam [58], or haloperidol [59]. The total number of patients in treatment comparison was 166. Delirium assessment was performed using validated tools (Table 1).

**Table 1:** Study characteristics of all included RCTs

	Study	Study location	Study date	Population	Procedure	Control medication	Total number of patients	Mean age (years)	Male (%)	Delirium assessment	Endpoint delirium
Sedation comparison	Chang [52]	Taiwan	2014–2015	Non-cardiac surgery	Postoperatively for a maximum of 24 h, RASS-2-0	Propofol	60	70.5	58.3	CAM-ICU	2
	Lee [55]	South Korea	2016–2017	Non-cardiac surgery	Intraoperatively as continuous infusion or bolus	Placebo	318	73	44.3	CAM	1
	Mei [54]	China	2016–2017	Non-cardiac surgery	Intraoperatively as bolus and subsequent continuous infusion	Propofol	296	75	45.6	CAM	1
	Sheikh [53]	India	2014–2016	Cardiac surgery	Intraoperatively as bolus and subsequent continuous infusion	Propofol	60	34.6	N/A	*	2
	Skrobik [56]	USA	2013–2016	MV**	9:30 in the evening until 6:15 the next morning, continuous infusion without bolus, RASS-1	Placebo	100	62.3	64	ICDSC	1
	Deiner [33]	USA	2008–2014	Non-cardiac surgery	Entering OR until 2 h in recovery room	Placebo	390	74***	48.7	CAM CAM-ICU	1
	Kawazoe [44]	Japan	2013–2016	MV	Continuously until end of MV, RASS-0 by day, RASS-2 by night	Propofol midazolam	201	68.5	63.2	CAM	2
	Li [45]	China	2014–2015	Cardiac surgery	Intraoperatively and continuously until end of MV	Placebo	285	67	69.1	CAM CAM-ICU	1
	Li [43]	China	2015–2016	MV	Sedation for at least 48 h until end of MV, RASS-0–1 in intervention, RASS − 2/− 3 in control group	Propofol, midazolam	126	44	56.3	CAM-ICU	1
	Djaiani [46]	Canada	2011–2014	Cardiac surgery	Postoperatively and continuously until end of MV	Propofol	183	72.6	75.4	CAM CAM-ICU	1
	Liu [47]	China	2015	Cardiac surgery	Postoperatively on ICU admission and continuously until end of MV, RASS-3-0	Propofol	61	54***	41	CAM	2
	Liu [49]	China	2014–2016	Non-cardiac surgery	Intraoperatively until 20 min before end of surgery	Placebo	197	73	48.7	CAM	1
	Su [58]	China	2011–2013	Non-cardiac surgery	Postoperatively until 8:00 the next morning	Placebo	700	74.4	60.4	CAM	1
	MacLaren [50]	USA	2009–2012	MV	Continuously until end of MV, according to desired SAS	Midazolam	23	58.1	56.5	ICDSC	2
	Priye [51]	India	2012–2013	Cardiac surgery	Postoperatively for 12 h	Placebo	64	43.3	51.6	RASS	2
	Yang [34]	China	2013	Non-cardiac surgery	Intraoperatively until 1 h before end of surgery and postoperatively until 6:00 the next morning	Placebo	79	50.5	53.2	CAM-ICU	2
	Devlin [35]	USA	2008–2012	NIV	2 h stable after NIV mask removal, SAS 3–4	Placebo	33	65	51.5	ICDSC	2
	Park [36]	South Korea	2012–2013	Cardiac surgery	Postoperatively until end of MV, RSS 2–3	Remifentanil	142	52.7	55.6	CAM-ICU	1
	Huang [37]	China	2008–2011	NIV	Until end of sedation, RSS 2–3	Midazolam	62	64.5	41.9	N/A	2
	Jakob [38]	Europe + Russia	2007–2010	MV	Continuously until end of MV, RASS-3-0	Propofol, midazolam	998	65***	65.5	CAM-ICU	2
	Maldonado [39]	USA	N/A	Cardiac surgery	Postoperatively until end of MV	Propofol, midazolam	118	57.7	63.6	CAM CAM-ICU	1
	Ruokonen [40]	Switzerland + Finland	N/A	MV	Continuously until end of MV, according to desired RASS	Propofol, midazolam	85	66***	82.4	CAM-ICU	2
	Shehabi [41]	Australia	2004–2007	Cardiac surgery	Postoperatively until ICU discharge, MAAS 2–4	Morphine	299	71.3***	75.3	CAM-ICU	1
	Pandharipande [32]	USA	2004–2006	MV	Continuously until end of MV, according to desired RASS	Lorazepam	103	59.5***	51.5	CAM-ICU	1
	Corbett [42]	USA	2002–2004	Cardiac surgery	Postoperatively until end of MV, RSS 5 the first 2 h, RSS 3–4 afterward	Propofol	89	63	82	N/A	3
Treatment comparison	Reade [57]	Australia + New Zealand	2011–2013	AD (MV)	According to desired RASS = 0	Placebo	74	57.3***	75.7	CAM-ICU	3
	Yapici 2010	Turkey	2005–2007	AD (cardiac surgery)	According to blood pressure and heart rate	Midazolam	72	60	37.5	CAM-ICU	3
	Reade [59]	Australia	2006–2008	AD (MV)	According to desired RASS = 0	Haloperidol	20	60.3***	85	ICDSC	3

Incidence comparison, *normal matter*; treatment comparison, *italic matter*

*AD* agitated delirium, *CAM* confusion assessment method, *CAM-ICU* confusion assessment method for intensive care unit, *h* hours, *ICDSC* intensive care delirium screening checklist, *MAAS* Motor Activity Assessment Scale, *MV* mechanical ventilation, *N/A* not available, *NIV* noninvasive ventilation, *OR* operating room, *RASS* Richmond Agitation and Sedation Scale, *RSS* Ramsay Sedation Scale, *SAS* Riker Sedation and Agitation Scale

1, primary endpoint; 2, secondary endpoint; 3, no specific endpoint

*Delirium defined as shourt course illusions, confusion, and cerebral excitement in the postoperative period; **Most; ***Median age

Further information on the drug doses used throughout the studies is provided in Additional file 2: Table S4.

### Quality of evidence

The Cochrane Collaboration’s risk of bias assessment is provided in Table 2. Six [56] articles consisting of seven RCTs in the incidence comparison had low risk of bias [33–35, 38, 45]. Nine RCTs had an unclear risk of bias in at least one domain due to poor reporting (1808 patients) [32, 36, 37, 40, 41, 48, 50, 51, 53], and ten RCTs had a high risk of bias in at least one of the five assessed domains [39, 42–44, 46, 47, 49, 52, 54, 55]. In the treatment comparison, there was one RCT with low risk of bias [57], one with unclear risk of bias in all five domains [58], and one with high risk of bias in two of the five domains [59].

**Table 2:**
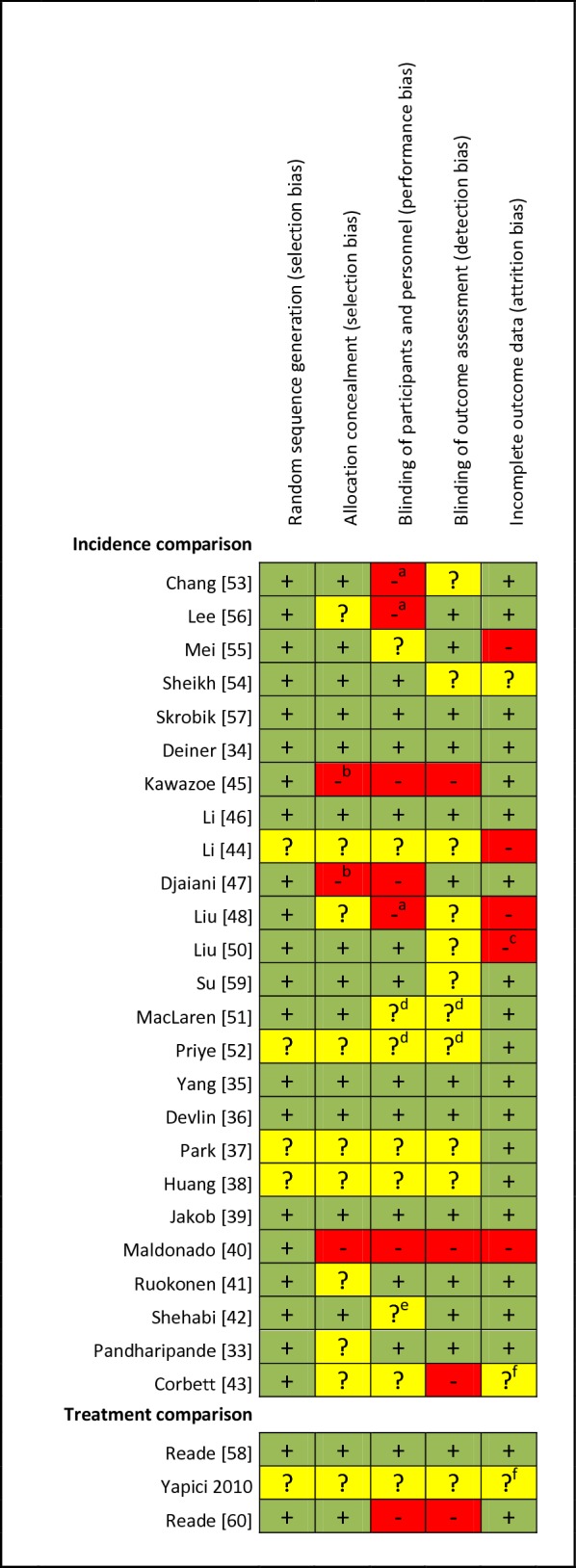
Risk of bias assessment of included RCTs

^a^Patient blinded

^b^Blocked randomization in unblinded trial

^c^Patients with delayed recovery were excluded

^d^Study reported as double blind; it is likely that patients and personnel were blinded, but this is unclear for the outcome assessor

^e^Study reported as double blind, but blinding of patients is not explicitly mentioned

^f^No flow chart provided; unclear if more patients were originally included

### Incidence comparison

Nine articles consisting of ten data sets compared dexmedetomidine to placebo; 14 articles consisting of 15 data sets compared dexmedetomidine to standard sedatives; and two RCTs (two data sets) compared dexmedetomidine to opioids (Table 3).

**Table 3:** Subgroup and sensitivity analyses for incidence of delirium

Subgroup and sensitivity analyses	Number of data sets	Number of patients	Risk ratio (95% CI) random effect model	*I* ^2^
*Placebo*				
Overall	10	2071	0.52 (0.42–0.63)	37%
Studies with no risk of bias according to our risk of bias assessment	5	887	0.66 (0.43–1.03)	20%
Cardiac surgery	2	349	0.53 (0.22–1.24)	1%
Mechanical ventilation	0			
Non-cardiac surgery	6	1589	0.53 (0.35–0.80)	61%
*Standard sedatives*				
Overall	15	2463	0.63 (0.46–0.86)	69%
Studies with no risk of bias according to our risk of bias assessment	2	998^a*^	0.64 (0.41–0.98)	0%
Cardiac surgery	5	509	0.40 (0.23–0.69)	11%
Mechanical ventilation	7	1536	0.82 (0.60–1.11)	66%
Non-cardiac surgery^b^	2	356	0.46 (0.23–0.90)	NA
Trim-and-fill method	20^c^	–	0.77 (0.57–1.05)	64%
*Opioids*				
Overall	2	441	0.61 (0.44–0.83)	0%

^a^Propofol or midazolam was used in the comparison group

^b^One out of two studies had no events

^c^Five hypothetical studies added with by a trim-and-fill function or R package meta

#### Incidence of delirium

**Fig. 2:**
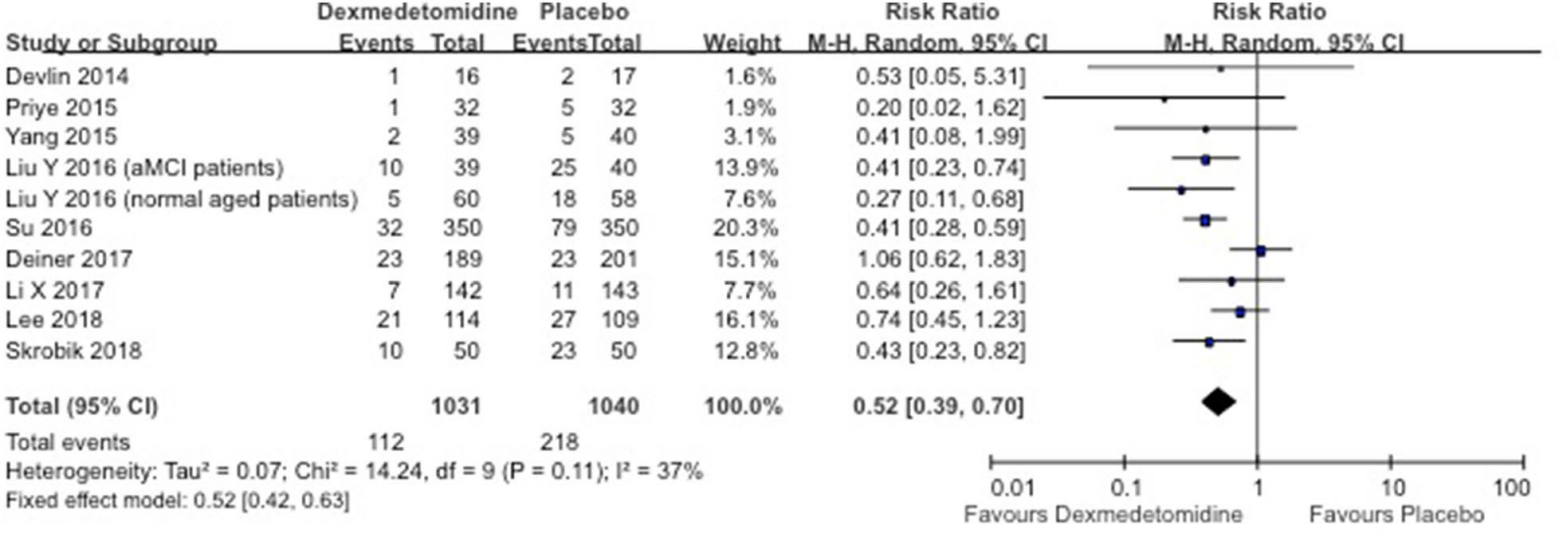
Forest plot for incidence of delirium in placebo-controlled RCTs. *aMCI* amnestic mild cognitive impairment

**Fig. 3:**
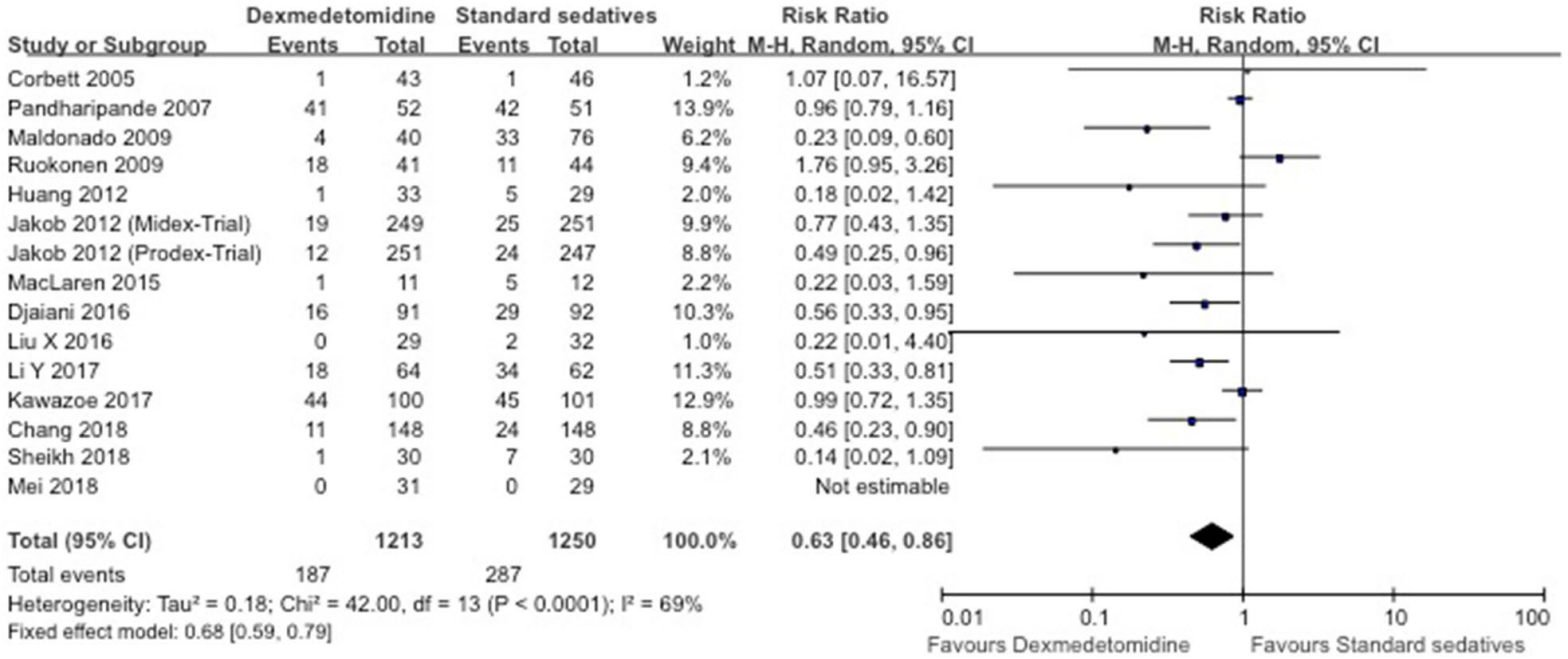
Forest plot for incidence of delirium in standard sedative-controlled RCTs

**Fig. 4:**
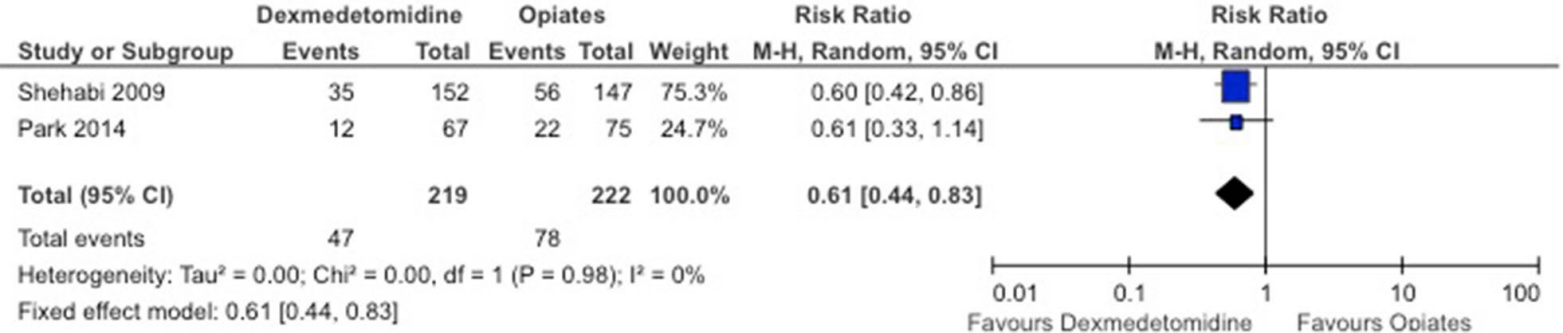
Forest plot for incidence of delirium in opioid-controlled RCTs

Overall incidence of delirium in the dexmedetomidine group was significantly lower compared to placebo (RR 0.52; 95% CI 0.39–0.70; *I*^2^ = 37%; Fig. 2), compared to standard sedatives (RR 0.63; 95% CI 0.46–0.86; *I*^2^ = 69%; Fig. 3), as well as compared to opioids (RR 0.61; 95% CI 0.44–0.83; *I*^2^ = 0%; Fig. 4). When only RCTs with a low risk of bias were included, we retrieved a RR of 0.66 (95% CI 0.43–1.03; five data sets included) for the placebo comparison and a RR of 0.64 (0.41–0.98; two data sets included) for the standard sedative comparison (Table 3). It is important to mention that the two data sets from the standard sedatives comparison were presented in the same article [38]. In the opioids group, there was no RCT without risk of bias.

Visual inspection of the funnel plot from the analysis of standard sedatives reveals some deviation from the funnel shape (Additional file 1: Figure S1), indicating the presence of publication bias (Egger’s test: *p* = 0.015). This was, however, not the case for the placebo comparison (Egger’s test: *p* = 0.62; Additional file 1: Figure S2).

Results from the trim-and-fill method (dexmedetomidine vs. standard sedatives) were less clear about the favorable effect compared to the overall result (RR 0.77; 95% CI 0.57–1.05; Table 3). Further subgroup and sensitivity results are presented in Table 3. Considerable heterogeneity was found in the subgroup analysis.

#### Adverse events

Explorative analysis of adverse events raising no claim for completeness is presented in Additional file 1: Table S5. When compared to placebo, dexmedetomidine sedation showed a RR of 0.92 for mortality (95% CI 0.51–1.64; *I*^2^ = 0%) [33, 35, 45, 48, 56] and a RR of 0.80 for circulatory insufficiency (95% CI 0.32–2.00; Additional file 1: Table S5) [48]. The risks of bradycardia [33–35, 45, 48, 56] and hypotension [33–35, 45, 48, 56] were significantly higher in the dexmedetomidine group, as opposed to tachycardia [35, 48] and hypertension [33, 48].

When compared to standard sedatives, only risks of bradycardia (RR 2.05; 95% CI 1.31–3.22; *I*^2^ = 36%) [32, 37, 38, 40, 44, 49, 50, 52] and hypotension (RR 1.26; 95% CI 1.04–1.54; *I*^2^ = 9%) [37, 38, 40, 42, 47, 50, 52] were significantly higher in the dexmedetomidine group.

When compared to opioids, dexmedetomidine showed a RR of 0.48 for mortality (95% CI 0.09–2.60) [41] and a higher risk of bradycardia with a RR of 2.03 (95% CI 1.07–3.83; *I*^2^ = 22%) [36, 41].

#### Clinical outcomes

Explorative analysis of clinical outcomes raising no claim for completeness is presented in Additional file 1: Table S6. Two outcome measures (i.e., ICU length of stay and time to extubation or duration of mechanical ventilation) were estimated for dexmedetomidine compared to placebo, standard sedatives, and opioids with overall results in favor of dexmedetomidine (Additional file 1: Table S6).

### Treatment comparison

Three RCTs were identified, which reported different clinical outcomes (Additional file 1: Table S7). Control drugs were placebo [57], midazolam [58], and haloperidol [59], each in one RCT. Comparing dexmedetomidine to placebo, Reade et al. [59] found a significantly accelerated resolution of delirium in patients receiving dexmedetomidine (median, 23.3 h; *n* = 39 vs. 40.0 h; *n* = 32; median difference between groups, 16.0 h [95% CI 3.0–28.0 h]; *P* = 0.01). The risk of bias according to our assessment was low in this study. Using midazolam as control medication, Yapici et al. (2011) who assessed delirium based on CAM-ICU at 36 h and 60 h postoperatively had an unclear risk of bias in all five domains. While after 36 h all patient still had a delirium (i.e., 37 dexmedetomidine treated and 34 midazolam treated patients), they stated significantly lower number of patients with delirium in the intervention group 60 h postoperatively (1/37 patients; 2.7% vs. 7/34 patients; 21%; *P *< 0.05). Comparing dexmedetomidine to haloperidol, Reade et al. [59] measured proportion of time with satisfactory ICDSC below 4 and desirable ICDSC below 1 as clinical outcome. Patients who received dexmedetomidine “tended to spend a greater proportion of time with satisfactory scores” (median [IQR], 95.5% [51–100%] vs. 31.5% [17–97%]; *P *= 0.122 and 61% [0–100%] vs. 0% [0–0%]; *P *= 0.134, respectively) [59]. The RCT was a non-blinded pilot study (including 10 patients in each treatment arm) which had to goal to assess the feasibility of the trial design and the safety of the two treatments.

## Discussion

This meta-analysis suggests dexmedetomidine to be an efficient and reliable sedative agent to reduce the incidence of delirium in critically ill patients when compared to placebo, standard sedatives, and opioids. Our results indicate that the overall incidence of delirium in the dexmedetomidine group was significantly lower compared to placebo, standard sedatives, and opioids. The 2009 study by Maldonado et al. [39] proposed two theories to explain the decreased rates of delirium associated with sedation of dexmedetomidine: The first theory is based on the intrinsic delirium-sparing property determined by multiple characteristics of dexmedetomidine. Since GABA, the primary inhibitory neurotransmitter in the central nervous system seems to play a key role in the pathogenesis of delirium, it is plausible that GABAergic agents such as benzodiazepines and propofol are strongly involved in the development and prolongation of delirium [60, 61]. The second theory is that dexmedetomidine-induced sedation provides a more natural sleep-like sedation pattern, which might reduce the risk of developing delirium [19, 62]. Furthermore, dexmedetomidine has very little effect on the cholinergic system, which is strongly linked to cognitive functions and the development of delirium [63]. Finally, when used as an alternative to sedation with GABAergic agents and/or opioids, dexmedetomidine may decrease delirium incidence.

When analyzing cardiac and non-cardiac surgery patients separately, the risk ratios for placebo and standard sedatives seem to be similar to the overall results. As the number of included data sets becomes small in all sensitivity-and subgroup analyses, the results have to be interpreted with caution.

Reduction of overall delirium risk by dexmedetomidine compared to standard sedatives confirms the findings from a meta-analysis conducted and published by Xia et al. [22]. Moreover, a 2014 meta-analysis by Pasin et al. [20] reported that dexmedetomidine significantly decreased delirium incidence when compared to control patients and in a subgroup analysis comparing dexmedetomidine to midazolam. A subgroup of RCTs assessing only cardiac surgery patients showed also results favoring dexmedetomidine. This finding supports Herr et al.’s [64] statement, suggesting a potentially positive effect of dexmedetomidine on the incidence of ICU delirium when compared to standard sedatives. The only article [38] with a low risk of bias included two data sets which have similar results when compared to the 12 included data sets.

When compared to opioids, the overall risk of delirium seems to be significantly lower favoring dexmedetomidine. The result has to be interpreted with caution because the opioid-controlled group in this meta-analysis consists of only two RCTs with unclear risk of bias [36, 41].

Of note, it can be interpreted from the subgroup and sensitivity analysis that all data sets point into the same direction, but there are not many studies of high level of evidence estimated by the five dimensions of the risk of bias assessment.

Our additional explorative meta-analyses assessing clinical outcomes confirmed that the use of dexmedetomidine significantly increased the risks of bradycardia and hypotension [32–38, 40–42, 44, 45, 47, 48, 50]. However, we had only limited data to assess the mortality risk or risk of circulatory insufficiency. Large uncertainties exist as indicated by wide 95% CIs. Here a comprehensive meta-analysis for this endpoint is warranted.

For the treatment of delirium, we identified only three RCTs including all different comparators. Therefore, it is difficult to draw any conclusion about the potential of dexmedetomidine in the treatment of delirium. This systematic review demonstrated the essential need for a standardized measurement for the clinical recovery from delirium. Otherwise, it is impossible to meta-analyze subsequent RCTs on this topic.

Several limitations in our study need to be acknowledged. First, since we focused on incidence of delirium as our primary outcome, we excluded all RCTs not containing delirium data. Of these studies, some might probably have evaluated adverse events and clinical outcome. Therefore, our meta-analysis of adverse events and clinical outcome raises no claim to completeness. Second, we only search in PubMed and CENTRAL for articles published in English. Therefore, it is possible that we missed relevant RCTs because they were published in other languages or published in journals which are not indexed in PubMed or CENTRAL. However, a recently published meta-epidemiological study showed that searching only PubMed and CENTRAL leads in the vast majority to the same conclusion (eventually with less certainty) when compared to more comprehensive Cochrane reviews [65]. Third, only seven out of 28 RCTs could be classified as low risk of bias RCTs. Fourth, there were many significant variations across all RCTs, potentially affecting their comparability. These include different ICU populations, severities of illness, and sedation protocols, leading to heterogeneity in drug doses and potentially influencing outcomes, or different durations of drug administration. Fifth, publication bias could not be assessed in one of the three main comparisons due to the limited number of studies [29]. The found effect for the comparison between standard sedatives and dexmedetomidine seemed to be affected by publication bias. Therefore, the results from the trim-and-fill method might be more valid. A further potential limitation, common in all sedation trials, is a masking of differences in sedation if opioids have been administered for clinical reasons [40].

Overall, our systematic review and meta-analysis are the first to focus on the role of dexmedetomidine in the prevention and treatment of the ICU delirium without making any restrictions concerning comparator drugs or types of ICU population. It uses a systematic approach to evaluate the hypothesis that dexmedetomidine is more effective than common therapeutic strategies for the treatment and prevention of delirium.

The results of our meta-analysis suggest that dexmedetomidine may be more efficient in reducing ICU delirium incidence than placebo standard sedatives and opioids. The evidence on treatment of ICU delirium with dexmedetomidine is limited, as only three trials with different comparators each were suitable for our investigation, and needs to be further investigated [66]. Additionally, it is necessary to standardize clinical outcomes in general, and especially in the treatment section to facilitate meta-analyses, thereby ensuring robust evidence.

## Additional files

**Additional file 1.** Detailed search strategy of electronic database search of PubMed and the Cochrane Central Register of Controlled Trials (CENTRAL).

**Additional file 2: Table S4.** Table of drug doses used throughout the analyzed studies.

## Abbreviations

CAM: confusion assessment method
CENTRAL: Cochrane Central Register of Controlled Trials
CI: confidence interval
GABA: gamma-amino-butyric acid
ICDSC: intensive care delirium screening checklist
ICU: intensive care unit
IQR: interquartile range
MV: mechanical ventilation
OR: odds ratio
PAD: pain, agitation, and delirium
RCT: randomized controlled trial
SD: standard deviation
