# Supplementary material for: Dexmedetomidine in prevention and treatment of postoperative and intensive care unit delirium: a systematic review and meta-analysis
Source: Ann Intensive Care. 2018 Sep 20;8:92. doi: 10.1186/s13613-018-0437-z (PMC6148680; doi:10.1186/s13613-018-0437-z)
Supplement: Supplementary file 1 — Additional file 1. Detailed search strategy of electronic database search of PubMed and the Cochrane Central Register of Controlled Trials (CENTRAL). [file 13613_2018_437_MOESM1_ESM.docx]

**Additional files 1: Appendix**

**Dexmedetomidine in Prevention and Treatment**

**of Postoperative and Intensive Care Unit Delirium**

**A Systematic Review and Meta-Analysis**

Julian Flükiger, MD^1*^, Alexa Hollinger, MD^1,2,3*^, Benjamin Speich, PhD^4,5^, Vera Meier^1^, Janna Tontsch^1^, Tatjana Zehnder^1^, Martin Siegemund, MD^1^

^1^Department for Anesthesia, Surgical Intensive Care, Prehospital Emergency Medicine and Pain Therapy, University Hospital Basel, Basel, Switzerland

^2^Department of Anaesthesiology, Burn and Critical Care Medicine, AP-HP, Saint Louis and Lariboisière University Hospitals, 2 rue Ambroise Paré, 75010 Paris, France

^3^Inserm 942 Paris, Biomarqueurs et maladies cardiaques, Hôpital Lariboisière - Bâtiment Viggo Petersen, 41, boulevard de la Chapelle, 75475 Paris Cedex 10, France

^4^Basel, Institute for Clinical **Epidemiology** and Biostatistics, Department of Clinical Research, University of **Basel** and University Hospital **Basel**, Basel, Switzerland

^5^Centre for Statistics in Medicine, Nuffield Department of Orthopaedics, Rheumatology and Musculoskeletal Sciences, University of Oxford, United Kingdom

*Both authors contributed equally to study design and writing of the manuscript.

**TABLE S1** Basis of clinical outcome results for placebo-controlled RCTs.

| **Dexmedetomidine vs. placebo** | |
| --- | --- |
|  |  |
| **ICU Length of Stay** | |
| Study | Values |
| Deiner 2017 | Median + IQR |
| Li X 2017 | Median + 95% CI |
| Su 2016 | Median + 95% CI |
| Devlin 2014 | Median + IQR |
|  |  |
| **Time to extubation or duration of MV** | |
| Study | Values |
| Li X 2017 | Median + 95% CI |
| Su 2016 | Median + 95% CI |
| Priye 2015 | Mean + SD |
| Devlin 2014 | Median + IQR |
| **Abbreviations:** CI, confidence interval, ICU, intensive care unit; IQR, interquartile range; MV, mechanical ventilation; SD, standard deviation | |

**TABLE S2** Basis of clinical outcome results for standard sedative-controlled RCTs.

| **Dexmedetomidine vs. standard sedatives** | |
| --- | --- |
|  |  |
| **ICU length of stay** | |
| Study | Values |
| Kawazoe 2017 | Median + IQR |
| Li Y 2017 | Mean + SD |
| Djaiani 2016 | Median + range |
| Huang 2012 | Mean + SD |
| Jakob 2012 | Median + IQR |
| Maldonado 2009 | Mean + SD |
| Ruokonen 2009 | Median + range |
| Pandharipande 2007 | Median + IQR |
| Corbett 2005 | Median + IQR |
|  |  |
| **Time to extubation or duration of MV** | |
| Study | Values |
| Li Y 2017 | Mean + SD |
| Djaiani 2016 | Median + range |
| Liu X 2016 | Median + IQR |
| Jakob 2012 | Median + IQR |
| Maldonado 2009 | Mean + SD |
| Ruokonen 2009 | Median + range |
| Corbett 2005 | Mean + SD |
| **Abbreviations:** ICU, intensive care unit; IQR, interquartile range; MV, mechanical ventilation; SD, standard deviation | |

**TABLE S3** Basis of clinical outcome results for opioid-controlled RCTs.

| **Dexmedetomidine vs. opioids** | |
| --- | --- |
|  |  |
| **ICU length of stay** | |
| Study | Values |
| Park 2014 | Mean + SD |
| Shehabi 2009 | Median + IQR |
|  |  |
| **Time to extubation or duration of MV** | |
| Study | Values |
| Park 2014 | Mean + SD |
| Shehabi 2009 | Median + IQR |
| **Abbreviations:** ICU, intensive care unit; IQR, interquartile range; MV, mechanical ventilation; SD, standard deviation | |

**TABLE S5** Adverse events in incidence comparison.

| **Adverse events** | **Dexmedetodimine vs. placebo** | | | | **Dexmedetodimine vs. standard sedatives** | | | | **Dexmedetodimine vs. opioids** | | | |
| --- | --- | --- | --- | --- | --- | --- | --- | --- | --- | --- | --- | --- |
|  | **Data sets (n)** | **Patients (n)** | **Random effects risk ratio** | ***I*^2^** | **Data sets (n)** | **Patients (n)** | **Random effects risk ratio** | ***I*^2^** | **Data sets (n)** | **Patients (n)** | **Random effects risk ratio** | ***I*^2^** |
| Mortality | 5 | 1508 | 0.92 (0.51-1.64) | 0% | 10 | 1907 | 0.97 (0.77-1.29) | 11% | 1 | 299 | 0.48 (0.09-2.60) | - |
| Bradycardia | 6 | 1587 | 1.35 (1.11-1.64) | 0% | 9 | 1829 | 2.05 (1.31-3.22) | 36% | 2 | 441 | 2.03 (1.07-3.83) | 22% |
| Tachycardia | 2 | 733 | 0.34 (0.30-0.76) | 0% | 4 | 1124 | 1.06 (0.70-1.62) | 75% | - | - | - | - |
| Hypotension | 6 | 1587 | 1.19 (1.03-1.38) | 0% | 8 | 1614 | 1.26 (1.04-1.54) | 9% | - | - | - | - |
| Hypertension | 2 | 1090 | 0.62 (0.49-0.80) | 0% | 2 | 998 | 1.18 (0.88-1.57) | 23% | - | - | - | - |
| Circulatory insufficiency | 1 | 700 | 0.80 (0.32-2.00) | - | - | - | - | - | - | - | - | - |

*Risk ratio for the following outcomes: mortality, bradycardia, tachycardia, hypotension, hypertension, circulatory insufficiency. Mean difference for the following outcomes: ICU length of stay (days), time to extubation or duration of mechanical ventilation (hours).

^a^Maldonado et al. 2009 had three treatment arms. Using the propofol- or the midazolam-arm in the control group did not significantly change the result for ICU length of stay (-1.23; 95% CI -2.17 to -0.29 and -1.24; 95% CI -2.24 to -0.23, respectively) and time to extubation or duration of mechanical ventilation (-5.53; 95% CI -10.45 to -0.62 and -6.75; 95% CI -12.16 to -1.33, respectively).

Abbreviations: CI = confidence interval; ICU=Intensive care unit.

**TABLE S6** Clinical outcomes of incidence comparison.

| **Clinical outcome** | **Dexmedetodimine vs. placebo** | | | | **Dexmedetodimine vs. standard sedatives** | | | | **Dexmedetodimine vs. opioids** | | | |
| --- | --- | --- | --- | --- | --- | --- | --- | --- | --- | --- | --- | --- |
|  | **Data sets (n)** | **Patients (n)** | **Random effects mean difference (95% CI)** | ***I*^2^** | **Data sets (n)** | **Patients (n)** | **Random effects mean difference (95% CI)** | ***I*^2^** | **Data sets (n)** | **Patients (n)** | **Random effects mean difference (95% CI)** | ***I*^2^** |
| ICU length of stay (days) | 5 | 1508 | -0.03 (-0.08 to 0.01) | 0% | 11 | 1983 | -1.29 (-2.15 to -0.42)^a^ | 82% | 2 | 441 | 0.03 (-0.26 to 0.33) | 0% |
| Time to extubation or duration of mechanical ventilation (hours) | 5 | 864 | -0.47 (-1.31 to 0.38) | 43% | 9 | 1618 | -3.95 (-7.61 to -0.28)^a^ | 81% | 2 | 441 | -0.01 (-4.92 to 4.90) | 49% |

^a^Maldonado et al. 2009 had three treatment arms. Using the propofol- or the midazolam-arm in the control group did not significantly change the result for ICU length of stay (-1.29; 95% CI -2.15 to -0.42 and -1.29; 95% CI -2.21 to -0.37, respectively) and time to extubation or duration of mechanical ventilation (-3.95; 95% CI -7.61 to -0.28 and -4.51; 95% CI -8.32 to -0.70, respectively).

**TABLE S7** Clinical outcomes of treatment comparison.

| **Study** | **Clinical outcome** | **Intervention** | **Control** | ***P*-value** |
| --- | --- | --- | --- | --- |
| **Reade 2016** | Resolution of delirium (hr), median (IQR) | 23.3 (13.0-54.0) | 40 (25.3-76.0) | 0.01 |
| **Yapici 2010** | CAM-ICU ≥ 3 36 hr postoperative, n | 38 of 38 patients | 34 of 34 patients | >0.05 |
|  | CAM-ICU ≥ 3 60 hr postoperative, n | 1 of 38 patients | 7 of 34 patients | <0.05 |
| **Reade 2009** | Proportion of time with ICDSC<4, %: median (IQR) | 95.5 (51-100) | 31.5 (17-97) | 0.122 |
|  | Proportion of time with ICDSC<1, %: median (IQR) | 61 (0-100) | 0 (0-0) | 0.134 |
| **Abbreviations:** hr, hours; ICDSC, Intensive Care Delirium Screening Checklist; IQR, interquartile range. | | | | |

**FIGURE S1** Funnel plot for standard sedative-controlled RCTs.


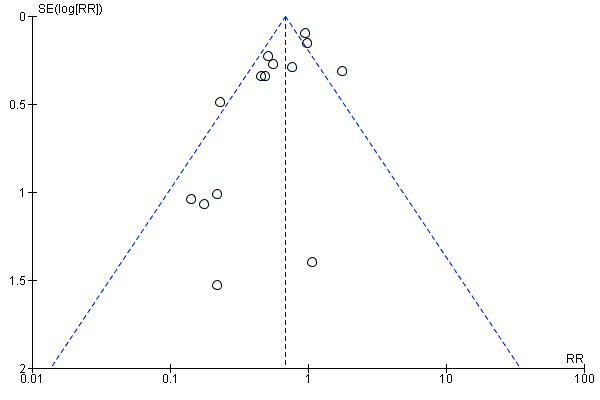


**FIGURE S2** Funnel plot for placebo-controlled RCTs


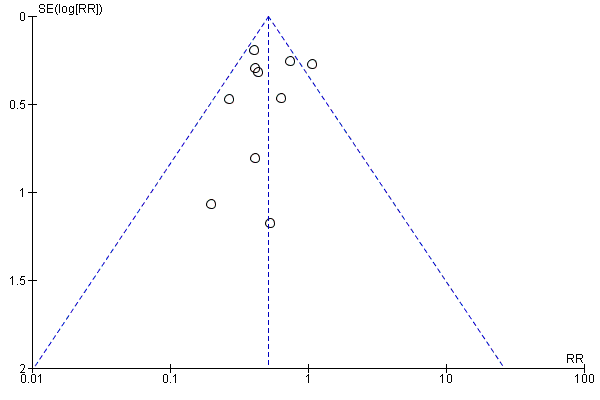


**SEARCH STRATEGY**

Detailed search strategy:

CENTRAL:

#1: MeSH descriptor: [Dexmedetomidine] explode all trees

#2: MeSH descriptor: [Delirium] explode all trees

#3: MeSH descriptor: [Respiration] explode all trees

#4: #2 or #3

#5: #1 and #4

PubMed:

((((delirium) AND dexmedetomidine)) OR ((dexmedetomidine[MeSH Terms]) AND ((Respiration, Artificial[MeSH Terms]) OR delirium[MeSH Terms]))) AND ((((randomized controlled trial[pt] OR controlled clinical trial[pt] OR randomized[tiab] OR placebo[tiab] OR clinical trials as topic[mesh:noexp] OR randomly[tiab] OR trial[ti] NOT (animals[mh] NOT humans [mh])))))
