# Supplementary figures and images for: Dexmedetomidine in prevention and treatment of postoperative and intensive care unit delirium: a systematic review and meta-analysis
Source: Ann Intensive Care. 2018 Sep 20;8:92. doi: 10.1186/s13613-018-0437-z (PMC6148680; doi:10.1186/s13613-018-0437-z)

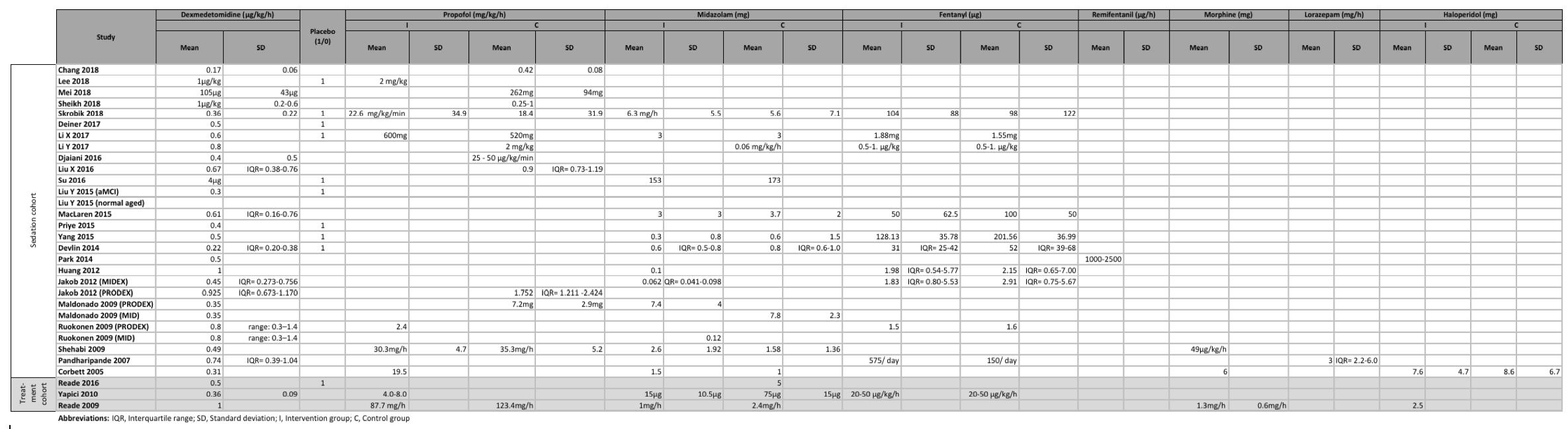

Supplement: Supplementary file 2 — Additional file 2: Table S4. Table of drug doses used throughout the analyzed studies. [file 13613_2018_437_MOESM2_ESM.png]
